# Interval walking training as a potential contributor to motor function improvement in adults with type 2 diabetes mellitus: a retrospective analysis

**DOI:** 10.3389/fendo.2025.1544831

**Published:** 2025-06-09

**Authors:** Masanori Yamazaki, Manami Hosokawa, Kohei Kitajima, Mitsuhisa Komatsu

**Affiliations:** Division of Diabetes, Endocrinology and Metabolism, Department of Internal Medicine, Shinshu University School of Medicine, Matsumoto, Japan

**Keywords:** type 2 diabetes mellitus, interval walking training, motor function, muscle quality, muscle mass

## Abstract

**Aim:**

In adults with type 2 diabetes mellitus (T2DM), hyperglycemia and related complications may impair skeletal muscle mass (SMM) and quality, leading to reduced motor function. This study aimed to evaluate the effects of interval walking training (IWT) on three motor function indicators: peak vertical ground reaction force normalized by body weight (F/w), rate of force development normalized by body weight (RFD/w), and balance index score (BIS).

**Methods:**

This retrospective analysis utilized data from a previous pilot trial of IWT. Changes in motor function were assessed using a motor function analyzer, and factors associated with these changes were identified using multiple linear regression analysis.

**Results:**

Among 51 adults with T2DM (including 24 aged ≥65 years), IWT significantly improved F/w (median [IQR]: 1.32 [1.26–1.36] to 1.32 [1.27–1.38] kgf/kg, p = 0.038), RFD/w (9.50 [8.03–13.12] to 10.2 [9.43–11.00] kgf/s/kg, p = 0.001), and BIS (52.0 [44.5–55.0] to 54.0 [48.0–56.0], p = 0.020). Notably, RFD/w showed significant improvement in both older (≥65 years: 9.45 [8.25–10.05] to 10.10 [8.80–10.45], p = 0.025) and non-older adults (<65 years: 9.90 [7.75–11.18] to 10.80 [9.58–11.85], p = 0.005). Baseline muscle quality was associated with changes in F/w and RFD/w, while increased leg SMM was linked to improvements in BIS.

**Conclusions:**

IWT may serve as a potential contributor to improved motor function in adults with T2DM, particularly when combined with strategies to maintain or enhance skeletal muscle quality and quantity.

## 1 Introduction

The increasing number of people with diabetes mellitus (DM) has become a critical global health issue. In 2021, 537 million adults aged 20–79 years in 215 countries and territories were estimated to have DM (1). In particular, with the rapid aging of the population, the number of older adults with DM increased to 13.9% of those aged 65 – 69 years in 2019. According to this estimate, the number of people older than 65 years with DM is projected to reach 195.2 million by 2030 (2).

DM is characterized by chronic hyperglycemic conditions that cause a high incidence of microangiopathies and atherosclerotic cardiovascular diseases (3). Musculoskeletal disorders are among the most common chronic complications of DM. Diabetic peripheral neuropathy, a major type of diabetic microangiopathy, has been implicated in the pathogenesis of diabetic muscle impairment (4). Additionally, hyperglycemia can directly and negatively affect muscle quality and quantity, leading to dynapenia, sarcopenia, and frailty. These clinical conditions share similar pathways with multiple pathological processes (5, 6). Sarcopenia and frailty can be further exacerbated by established classical diabetic complications, comorbidities, and various geriatric conditions, resulting in poor dynamic balance and increased physical disabilities (4, 5, 7). Muscle weakness resulting from sarcopenia and frailty often leads to slower movement and gait instability, which increases the risk of falls and hospitalization. These complications can diminish activities of daily living, reduce quality of life, and shorten life expectancy in individuals with DM (8, 9). Therefore, an effective strategy for addressing muscle health issues in individuals with DM is warranted.

Exercise is an essential treatment approach for individuals with DM. Exercise interventions, including both aerobic and resistance training, help alleviate hyperglycemia, reduce blood pressure, and improve blood lipid profiles (10). Furthermore, these training programs can assist individuals with DM in enhancing skeletal muscle strength and power, as well as improving weight control (11, 12). Balance training has been shown to lower the risk of falls, especially in older adults with type 2 diabetes mellitus (T2DM) (13). Based on these benefits, appropriate exercise prescriptions are likely to prevent skeletal muscle dysfunction and the deterioration of motor function.

In 1995, the Department of Sports Medical Sciences, Shinshu University Graduate School of Medicine, devised a unique exercise training program called ‘interval walking training (IWT)’ (14). The program includes repeated fast walking at ≥70% of the peak aerobic capacity (O_2_ peak) individually estimated by a specially manufactured triaxial accelerometer JD Mate (Kissei Comtec, Matsumoto, Japan) and slow walking at ≤40% of the O_2_ peak, alternately for 3 minutes each, 5–10 sets per day, and ≥4 days per week. Previous studies involving middle-aged and older individuals demonstrated that IWT effectively increased physical fitness, as evidenced by an increased O_2_ peak, and reduced lifestyle-related risk factors, including elevated blood glucose levels, blood pressure, body mass index (BMI), and body fat percentage, along with lower levels of blood high-density lipoprotein cholesterol (HDL-C) (14–16). IWT has also been shown to be effective in improving thigh muscle strength (15, 16). In adults with T2DM, IWT produced excellent results in improving physical fitness, body composition, and glycemic control, with high training adherence (17). However, these previous studies (15–17) did not adequately address the impact of IWT on not only muscle strength but also fundamental motor functions, including body stability during standing and moving, instantaneous force, and balance, in individuals with T2DM.

We hypothesized that IWT contributes positively to muscular endurance and neuromuscular function, providing benefits to adults with T2DM who are at risk for muscle impairment. This study aimed to evaluate the effectiveness of IWT in enhancing fundamental motor function. Additionally, it sought to identify physical and metabolic factors associated with improvements in motor functions among adults with T2DM.

## 2 Materials and methods

### 2.1 Study design and subjects

This study was retrospectively performed using data from a pilot intervention trial to evaluate the applicability of IWT as exercise therapy, the details of which have already been described (18). Briefly, the trial included 51 adults with T2DM who participated in a 20-week IWT program, aiming to train on more than four days per week, from September 2019 to May 2020. The inclusion criteria were: 1) a diagnosis of T2DM; 2) age between 20 and 80 years; 3) glycated hemoglobin (HbA1c) levels between 6.5 and 10.0%; 4) BMI between 20 and 34 kg/m^2^; and 5) the ability to perform exercise therapy in accordance with the regimen. The exclusion criteria included individuals with: 1) pre-proliferative or proliferative diabetic retinopathy; 2) diabetic nephropathy with macroalbuminuria (≥ 300 mg/gCre urinary albumin excretion); 3) an estimated glomerular filtration rate (eGFR), calculated by serum creatinine, ≤30 mL/min/1.73 m^2^; 4) preexisting coronary heart diseases or stroke; 5) pregnancy or lactation; or 6) any condition deemed inappropriate by the attending physician. The weekly target for fast walking time was set at ≥60 minutes. The intensity of fast walking was individually monitored using JD Mate devices to ensure it was performed at ≥70% of O2 peak. Training data collected by the JD Mate devices were transmitted to a central server via the internet. At each follow-up visit, participants received personalized exercise guidance through a remote individualized exercise prescription system, which provided detailed feedback on the previous two weeks of training and encouraged continued adherence. All participants completed the intervention. Individuals with motor function data both before and after the training were included in this analysis. This study was approved by the Institutional Review Board of the Shinshu University School of Medicine in accordance with the Declaration of Helsinki. Written informed consent was not required for this retrospective, single-site study.

### 2.2 Motor function and body composition analysis

**Figure 1:**
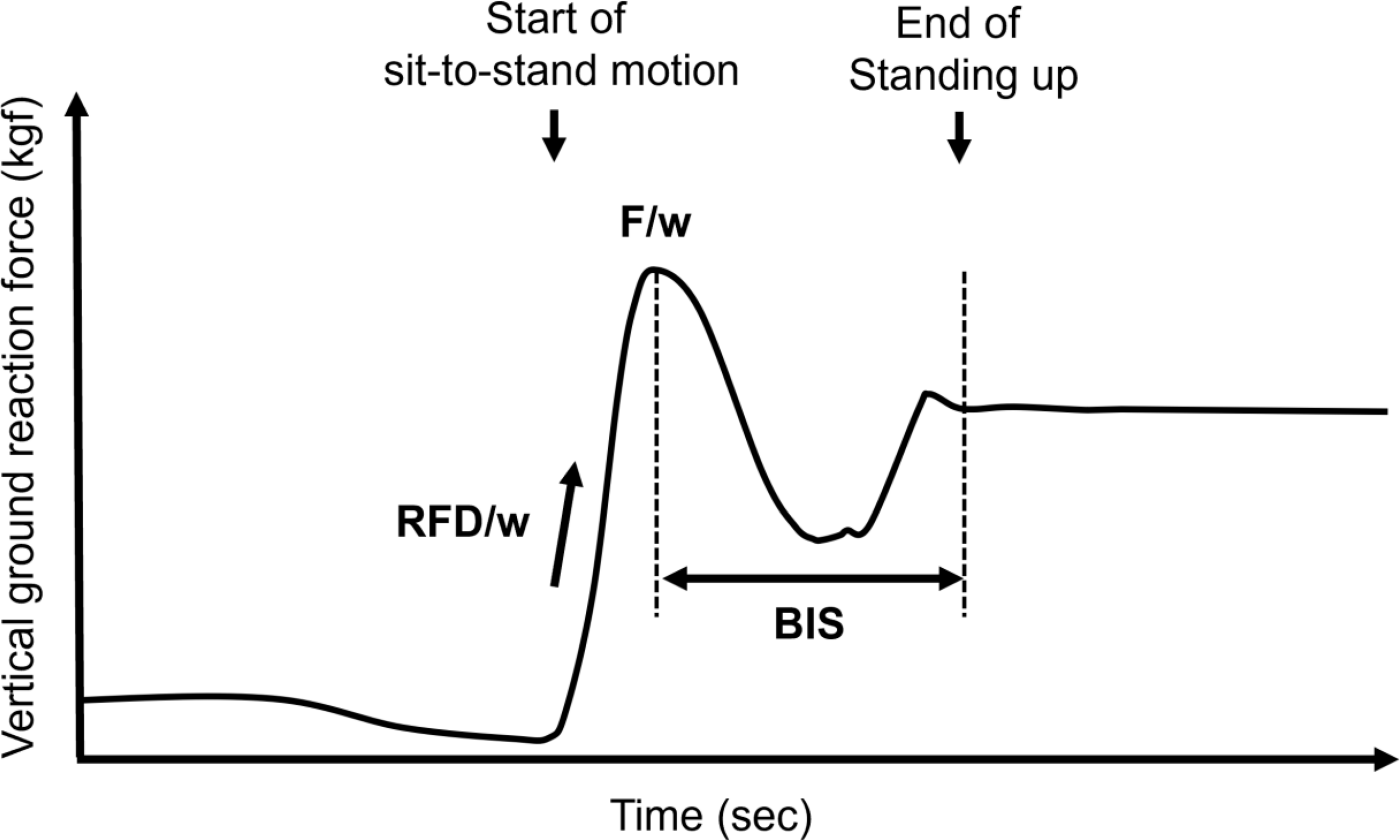
Definition of estimated motor functions during a stand-up motion.

Lower extremity motor function during the sit-to-stand movement was assessed using a motor function analyzer, zaRitz^®^ BT-220 (Tanita Corp., Tokyo, Japan), with a sampling frequency of 80 Hz. The assessment included measurements of peak vertical ground reaction force (GRF) (kgf) normalized by body weight (kg) (F/w); rate of force development (RFD) (kgf/s) normalized by body weight (kg) (RFD/w); and the balance index score (BIS). The BIS was calculated as the T-score of lateral load sway divided by the change in vertical load per second at the point of maximal RFD over 87.5 ms, normalized by body weight (kg) (**Figure 1**). In this analysis, the older units, kgf/kg and kgf/s/kg, were used for F/w and RFD/w, respectively, instead of the international system of units, Newton (N)/kg and N/s/kg, respectively. Previous studies have demonstrated that F/w, RFD/w, and BIS are correlated with handgrip strength, Timed Up & Go Test time, and single-leg standing time (19, 20). A higher BIS indicates greater movement amplitude and increased postural instability.

Participants were instructed to sit on a chair approximately 40 cm in height and stand up as quickly as possible. To standardize the assessment across participants, the starting position required a natural seated posture with the knees flexed at approximately 90° and feet placed on designated footprint markings on the analyzer platform, as initial foot placement can influence sit-to-stand performance. To eliminate the use of upper body momentum, participants were required to cross their arms over their chests and refrain from swinging their arms or using their hands to push off the chair.

A body composition analyzer (MC-780-A; Tanita Corp., Tokyo, Japan), employing bioelectrical impedance analysis, was used to evaluate total body fat mass, body fat percentage (total body fat mass normalized by body weight), skeletal muscle mass (SMM), skeletal muscle mass index (SMI) (appendicular skeletal muscle mass divided by the square of height), and a manufacturer-defined muscle mass quality score. The muscle mass quality score, ranging from 0 to 100, is calculated based on the ratio of muscle tissue cross-sectional area to total muscle cross-sectional area, derived from the electrical resistance of low- and high-frequency currents. Data on appendicular fat mass were unavailable.

### 2.3 Data collection

The following information, before and after IWT, was extracted from electronic medical records: age, duration of DM, existing diabetic complications, blood glucose-lowering prescriptions, height, weight, body composition, blood pressure, HbA1c, low-density lipoprotein cholesterol (LDL-C), HDL-C, triglycerides (TG), estimated glomerular filtration rate (eGFR), urinary albumin excretion rate (UACR), and motor function. BMI was calculated using height and weight. eGFR was determined using the Chronic Kidney Disease (CKD) Epidemiology Collaboration equation for Japanese individuals (21). Delta (Δ) was defined as the difference between the values after and before IWT.

### 2.4 Statistical analysis

No statistical sample size calculation was conducted. However, the actual sample size of 51 participants yielded a *post hoc* statistical power of 37.2% for F/w, 80.0% for RFD/w, and 50.6% for BIS to detect mean differences of 0.021 kgf/kg, 0.70 kgf/s/kg, and 2.2, respectively. These calculations were based on common standard deviations of 0.0089 kgf/kg, 0.174 kgf/s/kg, 0.78, respectively, using a paired two-sample t-test with a two-sided significance level of p < 0.05 to assess changes from baseline in each motor function parameter.

To identify factors associated with motor function improvement, participants were divided into two groups, based on whether or not they demonstrated increased function in each motor parameter. The term “improved group” referred to participants whose post-training measurements exceeded their corresponding pre-training values for a given motor function. Conversely, the “unimproved group” referred to participants whose post-training value was equal to or lower than the pre-training values.

Data are expressed as medians (interquartile range, IQR). Differences in measurement values before and after IWT were tested using the Wilcoxon signed-rank sum test (two-sided). The Mann-Whitney U test was used for group comparisons. Categorical values were compared using the χ^2^ test. Spearman’s rank correlation and univariate analyses were performed to assess the relationships between physical and biochemical continuous variables and changes in motor function before and after training. Stepwise multiple linear regression analysis was also conducted to identify independent factors associated with changes in each motor function indicator. All analyses were performed using StatFlex software (version 7.0; Arteck Inc., Osaka, Japan) or EZR software (version 1.68, Saitama Medical Center, Jichi Medical University, Saitama, Japan), a graphical user interface for R (The R Foundation for Statistical Computing, Vienna, Austria) (22). A p-value of <0.05 was considered statistically significant.

## 3 Results

### 3.1 Demographics of participants

The baseline characteristics of the study participants are summarized in **Table 1**. Among the 51 individuals who underwent the IWT, 24 (47.1%) were older adults (≥65 years, as defined by the World Health Organization), and the majority were male (29 individuals, 56.9%). The median age was 62.0 years (IQR, 54.0–72.8), and the median duration of DM was16 years (IQR, 10–23). Almost half of the candidates had diabetic neuropathy, with a significant higher prevalence observed in the older age group compared to the non-older (<65 years) age group (69.6 vs 26.9%, p = 0.003). The most prescribed antihyperglycemic agent was biguanide (metformin) (62.8%), followed by dipeptidyl peptidase 4 inhibitors (51.0%). Angiotensin-II receptor blockers and statins were administered to more than half of the participants. The median BMI and total time spent on fast walking were 26.4 kg/m^2^ (IQR 24.1–29.3) and 1022.0 min (IQR 590.3–1643.5), respectively.

**Table 1:** Participant demographics.

Characteristics	Overall group (N = 51)		Older age group (N = 24)		Non-older age group (N = 27)		p^†^
Age, years	62.0	(54.0–72.8)	73.0	(70.0–75.5)	54.0	(51.3–59.0)	<0.001
Male sex	29	(56.9)	17	(70.8)	35	(44.4)	0.058
Duration of DM, y	16	(10–23)	13.3	(13–25)	6.0	(6–18)	0.006
Current smoking habit	2	(4.0)	0	(0)	2	(7.4)	0.18
Current drinking habit	33	(64.7)	16	(66.7)	17	(63.0)	0.78
Diabetic neuropathy	23	(45.1)	16	(69.6)	7	(26.9)	0.003*
Use of hypoglycemic agents							
Glinides	3	(5.9)	2	(8.3)	1	(3.7)	0.46
Sulfonylureas	17	(33.3)	11	(45.8)	6	(22.2)	0.074
Biguanides	32	(62.8)	17	(70.8)	15	(55.6)	0.26
α-glucosidase inhibitors	10	(19.6)	6	(25.0)	4	(14.8)	0.29
Thiazolidine derivatives	1	(2.0)	1	(4.2)	0	(0)	0.47
DPP-4 inhibitors	26	(51.0)	14	(58.3)	12	(44.4)	0.32
SGLT 2 inhibitors	19	(37.3)	9	(37.5)	11	(40.7)	0.81
GLP-1 receptor agonists	13	(25.5)	8	(33.3)	5	(18.5)	0.23
Insulin	14	(27.5)	8	(33.3)	5	(18.5)	0.23
Use of anti-hypertensive agents							
Angiotensin-II receptor blockers	29	(56.9)	14	(58.3)	15	(55.6)	0.84
Calcium antagonists	22	(43.1)	10	(41.7)	12	(44.4)	0.84
α and β dual receptor blockers	5	(9.8)	2	(8.3)	3	(11.1)	0.56
Diuretics	6	(11.8)	2	(8.3)	4	(14.8)	0.39
Use of lipid-lowering agents							
Statins	25	(49.0)	12	(50.0)	13	(48.1)	0.89
Fibrates	2	(3.9)	0	(0)	2	(7.4)	0.28
SCAI	3	(5.9)	0	(0)	3	(11.1)	0.14
Eicosapentaenoic acid	1	(2.0)	0	(0)	1	(3.7)	0.52
Body mass index, kg/m^2^	26.4	(24.1–29.3)	24.4	(23.8–26.6)	28.2	(26.0–31.0)	0.002*
Total fast walking time, min	1022.0	(590.3–1643.5)	905.5	(581.0–1445.5)	1071.0	(606.5–1663.5)	0.62

Continuous variables are presented as median (interquartile range). Categorial variables presented as n (%). *Statistically significant (p <0.05) according to the Mann-Whitney U test. ^†^ Comparison between the groups aged ≥65 y and <65 y. DM, diabetes mellitus; DPP-4, dipeptidyl peptidase-4; SGLT2, sodium-glucose cotransporter 2; GLP-1: glucagon-like peptide-1; SCAI, selective cholesterol absorption inhibitor.

### 3.2 Influence of IWT on physical and metabolic parameters

The IWT-induced changes in physical parameters are presented in **Table 2** (overall group) and **Supplementary Table 2** (older vs. non-older adult age groups). Overall, body weight tended to increase 68.1 (IQR, 64.1–80.7) to 69.2 (IQR, 64.8–79.7) kg (p = 0.19). Total body fat mass significantly decreased from 22.4 (IQR, 16.5–29.0) to 22.2 (IQR, 16.8–28.0) kg (p = 0.016). Meanwhile, a slight increase in body fat percentage was observed from 31.1% (IQR, 22.5–39.5%) to 31.3% (IQR, 24.8–39.5%) (p = 0.029). These significant changes were also observed in both age groups. In older participants, total body fat mass decreased from 18.4 (IQR, 16.5–23.1) to 17.4 (IQR, 15.0–23.2) kg (p = 0.016), while body fat percentage increased from 26.7% (IQR, 23.4–36.6%) to 26.9% (IQR, 24.0–34.8%) (p = 0.029). In non-older participants, total body fat mass decreased from 26.2 (IQR, 21.4–32.5) to 25.2 (IQR, 15.0–23.2) (p = 0.003), and body fat percentage increased from 36.9% (IQR, 29.8–42.3%) to 37.1% (IQR, 26.4–41.5%) (p = 0.001). The muscle quality score increased from 51.0 (IQR, 41.0–63.8) to 55.0 (IQR 42.5–64.8) (p = 0.051). Regarding laboratory data, serum HDL-C significantly increased from 55.0 (IQR, 44.3–61.0 mg/dL) to 57.0 (IQR, 48.0–65.0) mg/dL overall (p = 0.014) and 54.0 (IQR, 44.5– 61.0) to 58.0 (IQR, 50.0–65.0) in the non-older age group (p = 0.012). In contrast, both LDL-C and casual triglyceride levels remained unchanged. HbA1c levels increased from 7.10% (IQR, 6.83–7.70%) to 7.30% (IQR, 7.00–7.78%) overall (p = 0.055).

**Table 2:** Changes in anthropometric measurements and laboratory data induced by interval walking training in the overall group (N = 51).

Characteristics	Pre-IWT		Post-IWT		Difference	p
Weight, kg	68.1	(64.1–80.7)	69.2	(64.8–79.7)	1.1	0.19
Total body fat mass, kg	22.4	(16.5–29.0)	22.2	(16.8–28.0)	-0.2	0.016*
Body fat percentage, %	31.1	(25.5–39.5)	31.3	(24.8–39.5)	0.2	0.029*
Muscle quality score	51.0	(41.0–63.8)	55.0	(42.5–64.8)	0.4	0.051
SMI, kg/m^2^	7.80	(7.10–8.40)	7.80	(7.13–8.70)	0	0.15
SMM
Whole body, kg	46.7	(38.1–49.9)	46.1	(38.3–50.5)	-0.6	0.46
Trunk, kg	25.6	(20.2–27.4)	25.4	(20.1–27.4)	-0.2	0.80
Left leg, kg	8.2	(7.0–9.1)	8.1	(7.0–9.1)	-0.1	0.58
Right leg, kg	8.0	(6.9–9.1)	8.2	(6.9–9.2)	0.2	0.37
Systolic blood pressure, mmHg	123.0	(112.3–130.8)	122.0	(116.0–130.0)	-1.0	0.56
Diastolic blood pressure, mmHg	76.0	(70.0–81.8)	78.0	(68.5–82.0)	2.0	0.33
HbA1c, %	7.10	(6.83–7.70)	7.30	(7.00–7.78)	0.20	0.055
eGFR, mL/min/1.73 m^2^	67.0	(58.0–79.0)	66.0	(55.3–74.0)	-0.1	0.26
LDL-C, mg/dL	98.0	(83.3–114.0)	104.0	(90.3–122.5)	6.0	0.16
HDL-C, mg/dL	55.0	(44.3–61.0)	57.0	(48.0–65.0)	2.0	0.014*
Casual triglycerides, mg/dL	141.0	(103.5–180.5)	146.0	(94.8–184.3)	5.0	0.14
UACR, mg/gCre	18.0	(8.0–41.8)	17.0	(8.0–43.0)	-1.0	0.52

Each value is represented as median (interquartile range). *Statistically significant (p <0.05) according to the Wilcoxon signed-rank sum test (two-sided). SMI, skeletal muscle index; SMM, skeletal muscle mass; eGFR, estimated glomerular filtration rate; LDL-C, low density lipoprotein cholesterol; HDL-C, high density lipoprotein cholesterol; UACR, urinary albumin creatinine ratio.

### 3.3 Enhancement of F/w, RFD/w, and BIS score through IWT

**Figure 2:**
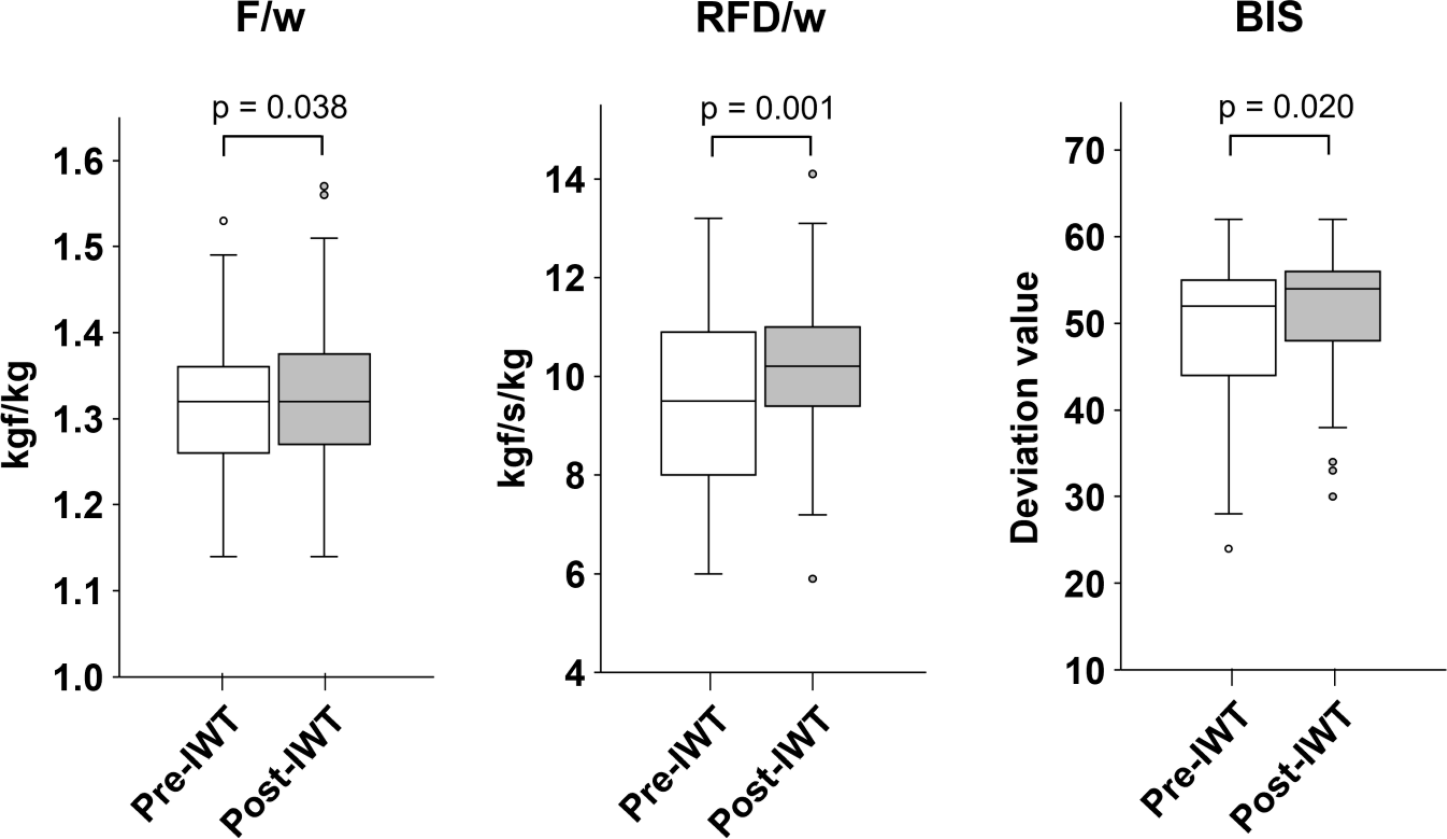
Change in motor functions following interval walking training in the overall group. Statical significance was determined using the Wilcoxon signed-rank sum test. In each box plot, the horizontal line within the box represents the median; the box extends from the 25th to the 75th percentile (interquartile, IQR); the vertical lines (whiskers) indicate adjacent values, defined as the most extreme values within 1.5 × IQR from the 25th and 75th percentiles; and dots represent outliers beyond the adjacent values.

**Figure 3:**
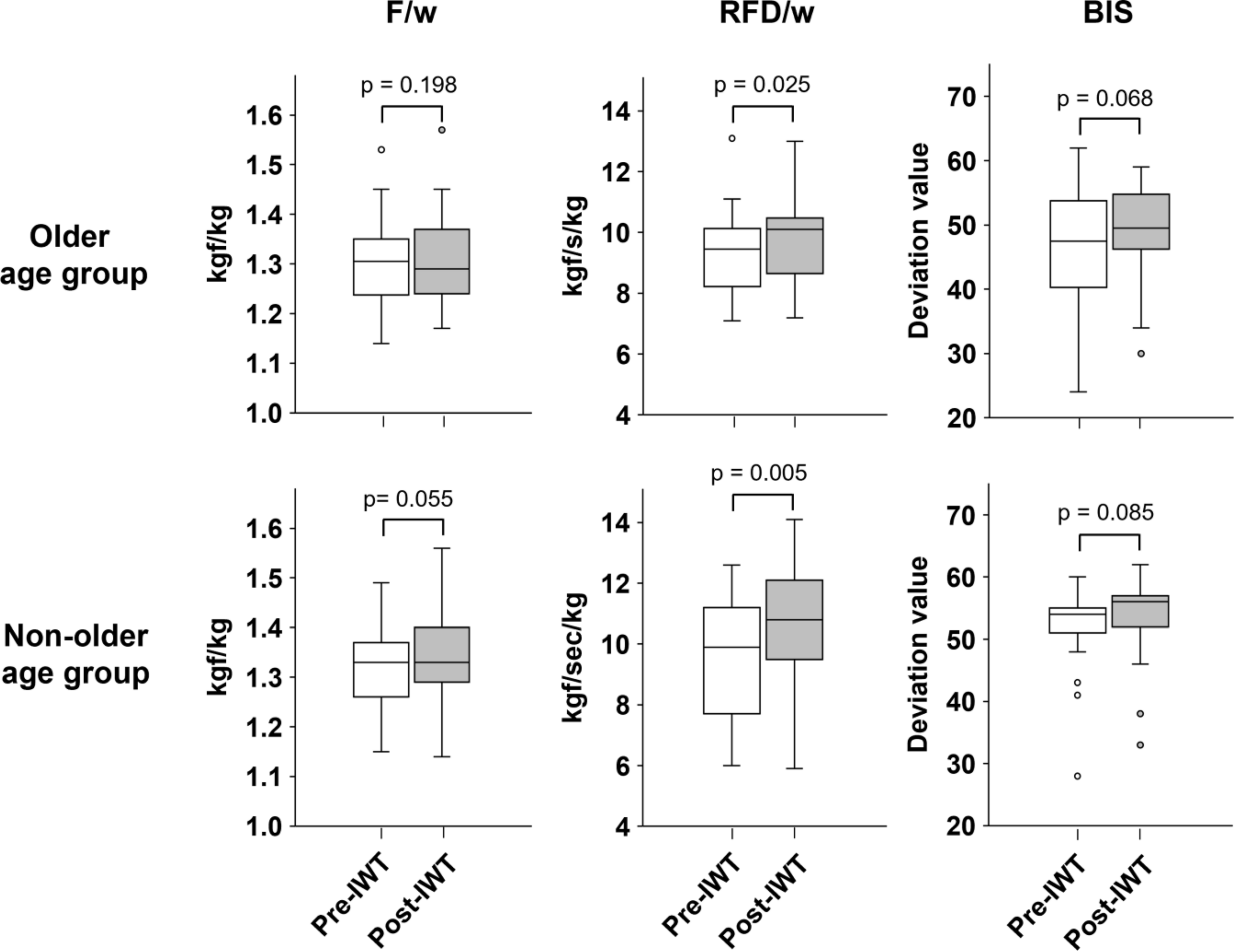
Change in motor functions induced by interval walking training in older and non-older age groups. Statical significance was determined using the Wilcoxon signed-rank sum test. In each box plot, the horizontal line within the box represents the median; the box extends from the 25th to the 75th percentile (interquartile, IQR); the vertical lines (whiskers) indicate adjacent values, defined as the most extreme values within 1.5 × IQR from the 25th and 75th percentiles; and dots represent outliers beyond the adjacent values.

We first examined IWT-induced changes in each motor function. The median (IQR) of F/w before and after IWT were 1.32 (1.26–1.36) and 1.32 (1.27–1.38) kgf/kg, respectively. This difference was statistically significant (p = 0.038). IWT also increased RFD/w from 9.50 (8.03–13.12) to 10.2 (9.43–11.00) kgf/s/kg (p = 0.001) and improved BIS from 52.0 (44.5–55.0) to 54.0 (48.0–56.0) (p = 0.020) (**Figure 2**). In both older and non-older age groups, IWT increased RFD/w (older age group: 9.45 [8.25–10.05] to 10.10 [8.80–10.45] kgf/s/kg, p = 0.025; non-older age group: 9.90 [7.75–11.18] to 10.80 [9.58–11.85] kgf/s/kg, p = 0.005). However, it did not significantly affect F/w (older age group: 1.31 [1.25–1.35] to 1.29 [1.24–1.37] kgf/kg, p = 0.198; non-older age group: 1.33 [1.26–1.37] to 1.33 [1.29–1.40] kgf/kg, p = 0.055) or BIS (older age group: 47.5 [40.5–53.5] to 49.5 [46.5–54.5], p = 0.068; non-older age group: 54.0 [51.0–59.7] to 56.0 [52.0–57.0] (p = 0.085) (**Figure 3**). These data indicate that IWT has the potential to enhance motor functions such as F/w, RFD/w, and BIS.

### 3.4 Comparison between the groups with and without F/w, RFD/w, and BIS

To identify significant factors related to motor function improvement, participants were divided into two groups: those with and those without improvements in each motor function, and their baseline characteristics and IWT-induced changes were compared. The results are shown in **Supplementary Table 2**, and the main differences are listed in **Table 3**. The F/w-improved group was less frequently treated with biguanides (48.0% vs. 76.9%, p = 0.033), insulin (12.0% vs. 38.5%, p = 0.030), and α- and β- dual receptor blockers (0 vs. 15.4%, p = 0.041). Additionally, the improved group had higher systolic blood pressure (128.0 vs. 120.0 mmHg, p = 0.020) and higher HDL-C levels (58.0 vs. 47.5 mg/dL, p = 0.025). Those who experienced improvement in BIS used fewer glinides (0% vs. 14.3%, p = 0.032) and had greater Δ SMI (0.10 vs. -0.10 kg/m^2^, p = 0.047) and Δ SMM in the right leg (0.10 vs. -0.10 kg, p = 0.036), in addition to a lower eGFR (65.0 vs. 71.0 mL/min/1.73 m^2^, p = 0.030). The analysis found no significant factors affecting RFD/w.

**Table 3:** Main differences in baseline characteristics and interval walking training-induced changes between groups with and without motor function improvements.

Motor function/characteristics	Improved group		Unimproved group		p
F/w					
N	25		26		
Biguanide	12	(48.0)	20	(76.9)	0.033*
Insulin	3	(12.0)	10	(38.5%)	0.030*
Calcium antagonists	7	(28.0)	14	(53.8)	0.061
α- and β- dual receptor blockers	0	(0)	4	(15.4)	0.041*
Systolic blood pressure, mmHg	128.0	(117.0–197.3)	120.0	(110.0–125.0)	0.020*
HDL-C, mg/dL	58.0	(49.0–64.5)	47.5	(46.0–57.0)	0.025*
Muscle quality score	58.0	(46.5–67.5)	47.5	(41.0–58.0)	0.051
RFD/w					
N	33		18		
Body fat percentage, %	33.7	(27.0–42.3)	29.9	(25.0–36.3)	0.073
BIS					
N	30		21		
Glinides	0	(0)	3	(14.3)	0.032*
eGFR, mL/min/1.73 m^2^	65.0	(53.0–71.0)	71.0	(64.0–80.0)	0.030*
Δ Weight	0.05	(-1.2–1.6)	-0.80	(-2.3–0.2)	0.060
Δ BMI, kg/m^2^	0	(-0.4–0.5)	-0.30	(-0.9–0.1)	0.067
Δ SMI, kg/m^2^	0.10	(-0.10–0.40)	-0.10	(-0.13–0.13)	0.047*
Δ SMM in the left leg, kg	0.10	(-0.2–0.4)	-0.10	(-0.3–0.2)	0.077
Δ SMM in the right leg, kg	0.10	(-0.1–60.4)	-0.10	(-0.2–0)	0.036*

Delta (Δ) represents the subtraction of pre-IWT value from post-IWT value of each item. *Statistically significant (p <0.05). F/w, the maximum ground reaction force based on weight; HDL-C, high density lipoprotein cholesterol; RFD/w, rate of force development based on weight; BIS, balance index score, BMI, body mass index; SMI, skeletal muscle index; SMM, skeletal muscle mass; eGFR, estimated glomerular filtration rate.

### 3.5 Significant factors related to IWT-induced changes in F/w, RFD/w, and BIS

Next, we performed a univariate analysis to identify specific factors associated with changes in motor function. The results are shown in **Table 4**. Δ F/w was weakly positively correlated with the baseline muscle quality score (ρ = 0.374, p = 0.007), systolic blood pressure (ρ = 0.394, p = 0.004), HDL-C (ρ = 0.309, p = 0.027), and F/w (ρ = -0.307, p = 0.028). Moreover, Δ F/w had a strong correlation with Δ RFD/w (ρ = 0.729, p <0.001). Δ RFD/w exhibited a weak correlation with baseline values of total body fat mass (ρ = 0.305, p = 0.030), F/w (ρ = -0.308, p = 0.028), and RFD/w (ρ = -0.386, p = 0.005). Δ BIS was weakly associated with a greater Δ SMM in the right leg (r = 0.324, p = 0.020) and strongly associated with a lower baseline BIS (ρ = -0.648, p <0.001).

**Table 4:** Significant factors associated with change in motor function in the overall group (N = 51).

Independent variables	Δ F/w, kgf/kg		Δ RFD/w, kgf/s/kg		Δ BIS	
	ρ	p	ρ	p	ρ	p
Age, years	-0.061	0.67	-0.165	0.25	0.124	0.39
Duration of DM, years	-0.105	0.47	-0.159	0.27	-0.137	0.34
Total fast walking time, min	-0.032	0.83	-0.021	0.89	0.263	0.062
Weight, kg	0.042	0.77	0.192	0.18	-0.046	0.75
Δ weight, kg	-0.007	0.96	0.008	0.95	0.263	0.062
BMI, kg/m^2^	0.118	0.41	0.233	0.10	-0.197	0.17
Total body fat mass, kg	0.146	0.31	0.305	0.030*	-0.156	0.28
Δ total body fat mass, kg	0.067	0.64	-0.021	0.88	-0.039	0.79
Body fat percentage, %	0.151	0.29	0.286	0.042*	-0.194	0.17
Δ body fat percentage, %	0.100	0.48	0.001	0.99	-0.156	0.28
Muscle quality score, points	0.374	0.007*	0.274	0.052	0.019	0.90
Δ muscle quality score, points	-0.063	0.66	0.066	0.64	0.026	0.86
SMI, kg/m^2^	-0.019	0.89	-0.037	0.79	0.063	0.66
Δ SMI, kg/m^2^	0.064	0.66	0.112	0.43	0.281	0.046*
SMM						
Whole body, kg	-0.079	0.58	-0.129	0.37	0.172	0.23
Δ whole body, kg	0.074	0.60	0.176	0.22	0.233	0.10
Trunk, kg	-0.062	0.66	-0.144	0.31	0.142	0.32
Δ trunk, kg	0.119	0.41	0.224	0.11	0.118	0.41
Left leg, kg	-0.007	0.96	-0.024	0.87	0.196	0.25
Δ Left leg, kg	0.053	0.71	0.090	0.53	0.244	0.084
Right leg, kg	-0.015	0.91	-0.033	0.82	0.163	0.25
Δ right leg, kg	0.050	0.73	0.117	0.42	0.324	0.020*
Systolic blood pressure, mmHg	0.394	0.004*	0.253	0.073	0.016	0.91
Δ systolic blood pressure, mmHg	-0.096	0.50	-0.168	0.23	0.041	0.78
Diastolic blood pressure, mmHg	0.222	0.12	0.081	0.57	0.100	0.49
Δ diastolic blood pressure, mmHg	-0.207	0.14	-0.148	0.30	0.071	0.62
HbA1c, %	-0.007	0.96	-0.037	0.80	-0.047	0.75
Δ HbA1c, %	0.287	0.041*	0.130	0.36	0.082	0.57
eGFR, mL/min/1.73 m^2^	0.015	0.91	0.036	0.80	-0.357	0.24
Δ eGFR, mL/min/1.73 m^2^	0.117	0.41	0.117	0.41	0.069	0.63
LDL-C, mg/dL	0.051	0.72	-0.002	0.99	-0.169	0.24
Δ LDL-C, mg/dL	0.264	0.061	0.202	0.16	0.078	0.58
HDL-C, mg/dL	0.309	0.027*	0.132	0.36	-0.189	0.18
Δ HDL-C, mg/dL	-0.144	0.31	-0.145	0.31	0.089	0.58
Casual triglyceride, mg/dL	-0.040	0.78	0.076	0.60	-0.040	0.78
Δ casual triglyceride, mg/dL	0.010	0.94	0.006	0.97	-0.087	0.54
UACR, mg/gCre	0.066	0.65	0.039	0.79	-0.019	0.90
Δ UACR, mg/gCre	0.065	0.67	0.001	1.0	-0.077	0.61
F/w	-0.307	0.028*	-0.308	0.028*	-0.045	0.76
RFD/w	-0.182	0.201	-0.386	0.005*	-0.080	0.58
Δ RFD/w	0.729	<0.001*	―	―	―	―
BIS	0.022	0.88	0.179	0.209	-0.648	<0.001*
Δ BIS	0.080	0.58	-0.048	0.74	―	―

Delta (Δ) represents the subtraction of pre-IWT value from post-IWT value of each item. *Statistically significant (p <0.05) according to the Spearman’s rank correlation. F/w, the maximum ground reaction force based on weight; RFD/w, rate of force development based on weight; BIS, balance index score; DM, diabetes mellitus; BMI, body mass index; HDL-C, high density lipoprotein cholesterol; eGFR, estimated glomerular filtration rate; SMI, skeletal muscle index; SMM, skeletal muscle mass; UACR, urinary albumin creatinine ratio.

Among the entire analytical data for older or non-older adults shown in **Supplementary Table 3**, several correlations were observed. In older adults, Δ F/w was moderately correlated with baseline systolic blood pressure (ρ = 0.548, p = 0.006) and Δ RFD/w (ρ = 0.607, p = 0.002). Δ RFD/w showed a weak correlation with Δ HDL-C (ρ = -0.448, p = 0.028) and a moderate correlation with baseline RFD/w (ρ = -0.414, p = 0.044). Δ BIS exhibited correlations with multiple variables, including duration of DM (ρ = -0.455, p = 0.029), Δ weight (ρ = 0.434, p = 0.034), and baseline values such as SMMs in the whole body (ρ = 0.461, p = 0.023), left leg (ρ = 0.491, p = 0.015), and right leg (ρ = 0.445, p = 0.029), as well as eGFR (ρ = -0.439, p = 0.032) and LDL-C (ρ = -0.407, p = 0.049). Meanwhile, in non-older adults, Δ F/w was associated with higher baseline values of body fat percentage (ρ = 0.408, p = 0.035), muscle quality score (ρ = 0.638, p <0.001), and Δ HDL-C (ρ = 0.398, p = 0.040). Delta RFD/w was correlated with age (ρ = -0.381, p = 0.049), baseline BMI (ρ = 0.423, p = 0.028), total body fat mass (ρ = 0.457, p = 0.017), body fat percentage (ρ = 0.442, p = 0.021), and muscle quality score (ρ = 0.514, p = 0.006). Factors related to Δ BIS were Δ SMI (ρ = 0.348, p = 0.075) and ΔSMM in the right leg (ρ = 0.408, p = 0.035).

For all participants, multiple linear regression analysis identified the following independent factors influencing each motor function: baseline systolic blood pressure (β = 0.002, p = 0.006), muscle quality score (β = 0.002, p = 0.003), and F/w (β = -0.233, p = 0.029) for Δ F/w; baseline muscle quality score (β = 0.042, p = 0.006), RFD/w (β = 0.575, p < 0.001), and BIS (β = 0.052, p = 0.031) for Δ RFD/w; and baseline eGFR (β = -0.134, p = 0.023), BIS (β = 0.453, p < 0.001), and Δ SMM in the right leg (β = 5.090, p = 0.048) for Δ BIS (**Table 5**).

**Table 5:** Independent factors associated with interval walking training-induced change in motor function.

Motor function	Variables	β	SE	t	p
Δ F/w^†^	Age	1.92 × 10^-4^	9.12 × 10^-4^	0.211	0.83
	Sex (female)	-0.001	0.019	-0.359	0.70
	Systolic blood pressure	0.002	6.32 × 10^-4^	2.869	0.006*
	Muscle quality score	0.002	7.83 × 10^-4^	3.188	0.003*
	F/w	-0.233	0.103	-2.252	0.029*
ΔRFD/w^‡^	Age	0.017	0.018	0.957	0.34
	Sex (female)	0.135	0.355	0.382	0.70
	Muscle quality score	0.042	0.015	2.862	0.006*
	RFD/w	0.575	0.093	6.156	< 0.001*
	BIS	0.052	0.023	2.228	0.031*
ΔBIS^§^	Age	-0.124	0.091	-1.367	0.179
	Sex (female)	-1.723	1.732	-0.995	0.325
	eGFR	-0.134	0.057	-2.362	0.023
	Δ SMM in the right leg	5.090	2.505	2.032	0.048*
	BIS	0.453	0.118	-3.824	< 0.001*

Delta (Δ) represents the subtraction of pre-IWT value from post-IWT value of each item. *Statistically significant (p <0.05) based on multiple linear regression analysis using the step-wise method. ^†^Adjusted R^2^ = 0.35. ^‡^Adjusted R^2^ = 0.38. ^§^Adjusted R^2^ = 0.41. F/w, the maximum ground reaction force based on weight; RFD/w, rate of force development based on weight; BIS, balance index score; BMI, body mass index; BMI, body mass index; HDL-C, high density lipoprotein cholesterol; eGFR, estimated glomerular filtration rate; SMI, skeletal muscle index; SMM, skeletal muscle mass; UACR, urinary albumin creatinine ratio.

## 4 Discussion

This study demonstrated that IWT enhanced three motor function indicators: F/w, RFD/w, and BIS. In both older and non-older age groups, IWT significantly improved RFD/w. Although F/w and BIS also exhibited an upward trend in both age groups, these changes did not reach statistical significance. To the best of our knowledge, this is the first study to report improvements in fundamental motor function induced by IWT.

In this study, IWT resulted in a significant improvement in F/w in the overall group; however, no significant changes were observed between the older and non-older age groups. This statistical discrepancy may be attributed to the smaller sample size within each subgroup. Previous studies have indicated that F/w reflects dynamic strength and power in the lower limbs (23, 24), with results comparable to or even exceeding those of the five-times STS test (24). In particular, a decline in F/w among older adults can lead to impaired balance due to muscle weakness, thereby increasing the risk of future falls (25, 26). Kera et al. (25) also found that reduced skeletal muscle strength associated with sarcopenia could be detected by identifying a lower power function, even without directly measuring skeletal muscle mass. This highlights muscle strength as a key factor in functional improvement. Muscle quality, often assessed by muscle density (i.e., the ratio of muscle mass to its volume), is also crucial for generating muscle strength. This is supported by findings from a cross-sectional study of 316 volunteers aged 59–85 years, in which muscle density showed a stronger association with muscle strength and physical performance, as assessed by the Timed Up and Go Test, than with muscle size (27). The importance of exercise training is further supported by evidence that continuous exercise improves muscle contraction dysfunction caused by impaired Ca^2+^ flux in skeletal muscles. This may lead to enhanced muscle quality and strength in the db/db mouse model of T2DM (28).

Baseline systolic blood pressure, muscle quality score, and F/w were markedly associated with increases in F/w in this study. This finding suggests that individuals with a lower baseline F/w may suggest greater improvements in F/w through IWT. The influence of blood pressure on F/w is considered to be mediated through its effect on muscle strength and quality. However, the mechanisms by which blood pressure affects muscle strength and quality are complex. Blachard et al. (29) reported that middle-aged, overweight adults in the early stages of hypertension, including systolic hypertension, exhibited greater muscle strength than those with normal blood pressure. This may be due to physical adaptations involving a shift in muscle fiber composition from Type I to Type IIb/x and a transition from oxidative to glycolytic metabolism. Similarly, Laddu et al. (30) found that higher grip strength was associated with higher systolic and diastolic blood pressure in older women. In contrast, Bai et al. (31) reported that muscle quality—defined as handgrip strength divided by lean soft tissue mass of the dominant arm—was negatively associated with hypertension prevalence and systolic blood pressure in adults aged 20−59 years. These findings suggest that the effect of blood pressure on muscle strength and quality may vary depending on characteristics of the study population, including age, sex, and the stages of hypertension. Our findings regarding the factors associated with increased F/w indicate that improvements in muscle strength may be strongly influenced by baseline muscle quality and strength. Therefore, in cases where conditions that impair muscle quality and strength—such as hyperglycemia (32) and malnutrition (33)—are present, addressing these underlying issues should be prioritized to facilitate improvement.

This study also demonstrated that IWT led to an improvement in RFD/w. The exact mechanism underlying this improvement remains unclear. One possible explanation is that increased lower limb strength may contribute to enhanced RFD. RFD, the primary component of RFD/w, is derived from the ascending part of the force-time curve of an explosive contraction, either as a mean time-locked value or as the maximal force-to-time ratio. RFD is considered more sensitive than maximal voluntary contraction force in detecting physiological changes induced by aging, immobilization, disuse, strength training, and rehabilitation (34). Moreover, RFD is influenced by both neuronal factors (such as neuronal drive, including motor unit firing rates) and muscular factors, including muscle fiber composition, stiffness of connective tissues including tendons, active cross-sectional area of muscles, and fast skeletal muscle contraction velocity (35, 36). Previous studies have reported that adults with DM exhibited increased Achilles tendon stiffness (37, 38), which may negatively affect walking performance. Considering these factors, the observed increase in peak GRF, as reflected by higher F/w, may be closely associated with enhanced lower limb strength, which in turn contributes to increased RFD/w.

In this study, baseline muscle quality score, RFD/w, and BIS were identified as significant positive factors for an increase in Δ RFD/w. This finding suggests that maintaining pre-IWT muscle quality, along with RFD/w and balance function, may be crucial for enhancing RFD/w through improved lower limb strength, which contributes to increases in F/w.

Maintaining better balance is essential for preventing falls and musculoskeletal injuries caused by unexpected shifts in the center of gravity, especially in the daily lives of older adults, with or without DM (39). This study revealed that BIS considerably improved through IWT in all participants, but not within each age group. Although this statistical discrepancy may be due to the smaller sample size in each group, IWT appears to have a greater tendency to enhance the balance in both groups.

The IWT-induced improvement in BIS was strongly associated not only with a higher baseline BIS but also with a lower baseline eGFR and enhanced SMM in the right leg. Our findings regarding the relationship between BIS and eGFR did not support previous reports suggesting that impaired renal function can lead to a decline in motor function including balance function (40, 41). Given the fact that serum creatinine levels are positively correlated with SMI (42), this discrepancy may be attributed to an elevation in serum creatinine levels induced by an increase in SMI. The growth and maintenance of SMM are also significantly important for improving balance function, as a cross-sectional study of 802 volunteers demonstrated a positive association between balance function and SMM (43). In fact, the present study showed that participants who exhibited an improvement in BIS experienced a greater increase in Δ SMI than those who did not. Additionally, a correlation was observed between an increase in SMM in the right leg, possibly the dominant foot, and the enhancement of BIS. Based on the results of the univariate analysis, this finding may be largely attributed to changes in SMM in the right leg among non-older adults. However, the influence of leg dominance on increases in SMM and balance function remains unclear in this study. Among individuals without specific conditions, such as athletes engaged in specific sports (44), differences in muscle mass between the dominant and non-dominant lower limbs have not been clearly established. Furthermore, a systematic review and meta-analysis reported that leg dominance does not significantly influence balance performance (45). Therefore, further research is necessary to elucidate the underlying mechanism of this phenomenon. To effectively support and maintain SMM for improved balance function, a combination of adequate protein supplementation and consistent resistance exercise training is strongly recommended (46).

The effect of IWT on glycometabolic profiles has been investigated in the pilot intervention trial linked to this study (18). The trial report indicated that IWT considerably increased serum HDL levels, while it did not improve HbA1c levels. The report suggested that the lack of improvement in HbA1c levels could be attributed to: 1) unregulated changes in the dosage of hypoglycemic agents and 2) seasonal fluctuations in blood glucose levels, which tend to be higher during the winter months at the trial site due to lower ambient temperatures.

A key strength of this study is its examination of the beneficial effects of IWT on fundamental motor function parameters in adults with T2DM. The findings from this study provide valuable evidence supporting the potential of IWT as an exercise therapy to help maintain motor function in individuals with T2DM. However, this study has some limitations. First, the study was conducted with a small sample size, which made it difficult to sufficiently identify motor-function-enhancing factors across the different age groups. Second, this study lacked a control group, which may have limited the ability to draw causal inferences. Third, this study was conducted as a retrospective study, which may have introduced potential selection biases and limited the ability to establish a clear causal relationship. Additionally, potential cofounding factors—such as actual daily physical activity levels, dietary habits, and changes in medication use that may affect muscle condition and function—could not be accounted for in the analysis due to the unavailability of detailed personal data. However, according to a report of the trial related to this study (18), there was no difference in daily energy intake or basal physical activity energy expenditure before and after the training, evaluated by brief-type self-administered diet history questionnaire (BDHQ) and measurement system JD mate (47). Taken together, future large-scale, multicenter, international randomized controlled trials are warranted to validate our findings and establish causality. Fourth, bioelectrical impedance analysis (BIA) may have limited sensitivity in detecting changes in body composition and is susceptible to measurement errors. Each parameter can be affected by the individual’s hydration status. Therefore, the absence of standardized measurement conditions and the lack of complementary methods—including computed tomography (CT), magnetic resonance imaging (MRI), and dual-energy X-ray absorptiometry (DXA)—may have compromised the accuracy of the results. Finally, supportive findings from a basic research perspective are lacking. Additional histological and biochemical examinations of skeletal muscles could provide valuable insights into the mechanisms underlying the beneficial effects of IWT on motor function improvement.

In conclusion, this study demonstrated that IWT considerably improved motor functions—including F/w, RFD/w, and BIS—in adults with T2DM. Notably, these improvements may be related to pre-IWT skeletal muscle mass and quality, as well as IWT-induced increases in lower limb muscle mass. IWT may thus serve as a potential contributor to improved motor function in adults with T2DM, particularly when combined with strategies to maintain or enhance skeletal muscle quality and quantity.

## Data Availability

The original contributions presented in the study are included in the article/**Supplementary Material**. Further inquiries can be directed to the corresponding author.
