# Supplementary material for: Interval walking training as a potential contributor to motor function improvement in adults with type 2 diabetes mellitus: a retrospective analysis
Source: Front Endocrinol (Lausanne). 2025 Jun 9;16:1544831. doi: 10.3389/fendo.2025.1544831 (PMC12183035; doi:10.3389/fendo.2025.1544831)
Supplement: Supplementary file 1 [file Table1.docx]

**Supplementary Table 1** Changes in anthropometric measurements and laboratory data induced by interval walking training in older and non-older age groups

| **Characteristics** | **Older age group (N = 24)** | | | | |  | **Non-older age group (N = 27)** | | | | |
| --- | --- | --- | --- | --- | --- | --- | --- | --- | --- | --- | --- |
|  | **Pre-IWT** | | **Post-IWT** | | **p** |  | **Pre-IWT** | | **Post-IWT** | | **p** |
| Weight, kg | 65.9 | (63.2–  71.7) | 66.6 | (61.8–  72.8) | 0.19 |  | 76.8 | (65.3–  85.9) | 77.0 | (65.7–  83.6) | 0.059 |
| Total body fat mass, kg | 18.4 | (15.6–  23.1) | 17.4 | (15.0–  23.2( | 0.016* |  | 26.2 | (21.4–  32.5) | 25.2 | (21.5–  32.5) | 0.003* |
| Body fat percentage, % | 26.7 | (23.4–  36.6) | 26.9 | (24.0–  34.8) | 0.029* |  | 36.9 | (29.8–  42.3) | 37.1 | (26.4–  41.5) | 0.001* |
| Muscle quality score | 46 | (38.5–  54.0) | 48 | (41.0–  56.5) | 0.051 |  | 61 | (48.0–  66.8) | 64 | (50.5–  80.0) | 0.068 |
| SMI, kg/m^2^ | 7.90 | (7.10–  8.25) | 7.80 | (7.20–  8.20) | 0.15 |  | 7.80 | (7.10–  8.80) | 7.90 | (7.13–  9.00) | 0.030* |
| SMM |  |  |  |  |  |  |  |  |  |  |  |
| Whole body, kg | 47.5 | (37.8–  48.9) | 47.0 | (38.3–  49.7) | 0.46 |  | 41.9 | (38.1–  54.2 | 43.0 | (38.3–  54.6) | 0.31 |
| Trunk, kg | 25.8 | (19.3–  26.6) | 25.8 | (19.8–  26.6) | 0.80 |  | 21.7 | (20.5–  29.2) | 22.1 | (20.3–  29.0) | 0.62 |
| Left leg, kg | 8.3 | (6.9–  8.9) | 8.1 | (6.8–  9.0) | 0.58 |  | 8.2 | (7.0–  9.7) | 8.3 | (6.9–  9.9) | 0.26 |
| Right leg, kg | 8.1 | (7.1–  9.0) | 8.2 | (6.9–  9.0) | 0.37 |  | 8.0 | (6.9–  9.6) | 8.0 | (6.8–  9.7) | 0.17 |
| Systolic blood pressure, mmHg | 126.0 | (118.5–  133.5) | 122.0 | (116.0–132.0) | 0.56 |  | 120.0 | (111.3–  129.5) | 122.0 | (110.5–  128.0) | 0.97 |
| Diastolic blood pressure, mmHg | 74.5 | (68.0–  78.0) | 74.5 | (68.0–  80.5) | 0.33 |  | 76.0 | (70.0–  84.0) | 79.0 | (72.3–  84.0) | 0.63 |
| HbA1c, % | 7.10 | (6.75–  7.65) | 7.40 | (7.05–  7.90) | 0.055 |  | 7.10 | (6.90–  7.70) | 7.30 | (6.85–  7.78) | 0.53 |
| eGFR, mL/min/ 1.73 m^2^ | 64.0 | (51.5–  71.5) | 64.0 | (53.5–  69.5) | 0.26 |  | 68.0 | (64.0–  89.0) | 71.0 | (63.0–  81.3) | 0.38 |
| LDL-C, mg/dL | 93.0 | (84.5–  112.0) | 115.5 | (92.5–  115.5) | 0.16 |  | 100.0 | (83.3–  118.5) | 110.0 | (81.5–  129.3) | 0.67 |
| HDL-C, mg/dL | 55.5 | (42.5–  62.0) | 53.5 | (46.5–  66.0) | 0.014* |  | 54.0 | (44.5–  61.0) | 58.0 | (50.0–  65.0) | 0.012* |
| Casual triglycerides, mg/dL | 124.5 | (98.5–  161.5) | 137.0 | (78.5–  178.0) | 0.14 |  | 167.0 | (111.3–  195.5) | 151.0 | (105.8–  187.3) | 0.12 |
| UACR, mg/gCre | 18.5 | (10.0–  76.0) | 17.0 | (8.5–  50.5) | 0.52 |  | 16.0 | (6.0–  36.5) | 17.0 | (7.0–  33.5) | 0.48 |

Each value is represented as median (interquartile range). *Statistically significant (p <0.05) according to the Wilcoxon signed-rank test (two-sided). IWT, interval walking training; SMI, skeletal muscle index; SMM, skeletal muscle mass; eGFR, estimated glomerular filtration rate; LDL-C, low density lipoprotein cholesterol; HDL-C, high density lipoprotein cholesterol; UACR, urinary albumin creatinine ratio
