# Supplementary material for: Interval walking training as a potential contributor to motor function improvement in adults with type 2 diabetes mellitus: a retrospective analysis
Source: Front Endocrinol (Lausanne). 2025 Jun 9;16:1544831. doi: 10.3389/fendo.2025.1544831 (PMC12183035; doi:10.3389/fendo.2025.1544831)
Supplement: Supplementary file 2 [file Table2.docx]

**Supplementary Table 2** Comparison between groups with and without motor function improvements

|  | **F/w** | | | | |  | **RFD/w** | | | | |  | **BIS** | | | | |
| --- | --- | --- | --- | --- | --- | --- | --- | --- | --- | --- | --- | --- | --- | --- | --- | --- | --- |
| **Characteristics** | **Improved**  **group**  **(N = 25)** | | **Unimproved group**  **(N = 26)** | | **p** |  | **Improved group**  **(N = 33)** | | **Unimproved group**  **(N = 18)** | | **p** |  | **Improved**  **group**  **(N = 30)** | | **Unimproved group**  **(N = 21)** | | **p** |
| Age, years | 63.0 | (51.5–75.3) | 61.5 | (55.0–71.0) | 0.97 |  | 61.0 | (53.8–  72.3) | 63.5 | (55.0–74.0) | 0.69 |  | 64.0 | (55.0–74.0) | 60.0 | (54.0–70.3) | 0.31 |
| Male sex | 11 | (44.0) | 11 | (42.3) | 0.90 |  | 16 | (48.5) | 13 | (72.2) | 0.10 |  | 11 | (36.7) | 11 | (52.4) | 0.26 |
| Current smoking habit | 0 | (0) | 2 | (7.7) | 0.17 |  | 31 | (93.9) | 17 | (94.4) | 0.67 |  | 27 | (90.0) | 21 | (100) | 0.22 |
| Current drinking habit | 18 | (72.0) | 15 | (57.7) | 0.29 |  | 11 | (33.3) | 7 | (38.9) | 0.69 |  | 2 | (6.7) | 0 | (0) | 0.40 |
| Duration of DM, years | 15.5 | (10.0–21.0) | 16.0 | (10.0–25.0) | 0.65 |  | 15.0 | (11.3) | 17.0 | (19.5) | 0.31 |  | 13.0 | (10.0–18.0) | 23.0 | (9.0–28.0) | 0.10 |
| Diabetic neuropathy | 14 | (56.0) | 9 | (34.6) | 0.19 |  | 15 | (45.5) | 8 | (44.4) | 0.79 |  | 16 | (53.3) | 7 | (33.3) | 0.16 |
| Use of hypoglycemic agents |  |  |  |  |  |  |  |  |  |  |  |  |  |  |  |  |  |
| Glinides | 1 | (4.0) | 2 | (7.7) | 0.58 |  | 2 | (6.1) | 1 | (5.6) | 0.94 |  | 0 | (0) | 3 | (14.3) | 0.064 |
| Sulfonylureas | 8 | (32.0) | 9 | (34.6) | 0.84 |  | 11 | (33.3) | 6 | (33.3) | 1.0 |  | 9 | (30.0) | 8 | (38.1) | 0.55 |
| Biguanides | 12 | (48.0) | 20 | (76.9) | 0.033* |  | 18 | (18.2) | 14 | (22.2) | 0.10 |  | 17 | (56.7 | 15 | (71.4) | 0.28 |
| α-glucosidase inhibitors | 7 | (28.0) | 3 | (11.5) | 0.14 |  | 6 | (28.0) | 4 | (11.5) | 0.73 |  | 6 | (20.0) | 4 | (19.1) | 0.61 |
| Thiazolidine derivatives | 1 | (4.0) | 0 | (0) | 0.49 |  | 0 | (0) | 1 | (5.6) | 0.17 |  | 1 | (3.3) | 0 | (0) | 0.59 |
| DPP-4 inhibitors | 13 | (52.0) | 13 | (50.0) | 0.89 |  | 18 | (54.5) | 8 | (44.4) | 0.49 |  | 15 | (50.0) | 11 | (52.4) | 0.87 |
| GLP-1 receptor agonists | 7 | (28.0) | 6 | (23.1) | 0.69 |  | 7 | (21.2) | 6 | (33.3) | 0.34 |  | 9 | (30.0) | 4 | (19.1) | 0.38 |
| SGLT2 inhibitors | 10 | (40.0) | 10 | (38.5) | 0.91 |  | 15 | (45.5) | 5 | (27.8) | 0.22 |  | 12 | (40.0) | 8 | (38.1) | 0.89 |
| Insulin | 3 | (12.0) | 10 | (38.5) | 0.030* |  | 7 | (21.1) | 6 | (38.5) | 0.34 |  | 9 | (30.0) | 4 | (19.1) | 0.38 |
| Use of anti-hypertensive agents |  |  |  |  |  |  |  |  |  |  |  |  |  |  |  |  |  |
| Angiotensin-Ⅱ receptor blockers | 14 | (56.0) | 14 | (53.8) | 0.88 |  | 20 | (60.6) | 9 | (50.0) | 0.46 |  | 17 | (56.7) | 11 | (52.4) | 0.76 |
| Calcium antagonists | 7 | (28.0) | 14 | (53.8) | 0.061 |  | 15 | (45.5) | 7 | (38.9) | 0.65 |  | 10 | (33.3) | 11 | (52.4) | 0.17 |
| α- and β- dual receptor blockers | 0 | (0) | 4 | (15.4) | 0.041* |  | 4 | (12.1) | 1 | (5.6) | 0.45 |  | 2 | (6.7) | 2 | (9.5) | 0.55 |
| Diuretics | 3 | (12.0) | 3 | (11.5) | 0.96 |  | 5 | (15.2) | 1 | (5.6) | 0.31 |  | 2 | (6.7) | 4 | (19.1) | 0.18 |
| Use of lipid lowering  drugs |  |  |  |  |  |  |  |  |  |  |  |  |  |  |  |  |  |
| Statin | 12 | (48.0) | 14 | (53.8) | 0.68 |  | 15 | (45.5) | 10 | (55.6) | 0.49 |  | 17 | (56.7) | 9 | (42.3) | 0.33 |
| Fibrate | 1 | (4.0) | 1 | (3.8) | 0.98 |  | 1 | (3.0) | 1 | (5.6) | 0.66 |  | 2 | (6.7) | 0 | (0) | 0.34 |
| Cholesterol absorption inhibitors | 1 | (4.0) | 2 | (7.7) | 0.58 |  | 1 | (3.0) | 2 | (11.1) | 0.24 |  | 2 | (6.7) | 1 | (4.8) | 0.64 |
| Eicosapentaenoic acid | 1 | (4.0) | 0 | (0) | 0.30 |  | 1 | (3.0) | 0 | (0) | 0.46 |  | 0 | (0) | 1 | (4.8) |  |
| Total fast walking time, min | 1026.0 | (616.3–  1494.8) | 959.0 | (575.0–  1362.0) | 0.81 |  | 914.0 | (613.3–1308.5) | 1055.5 | (587.0–1604.0) | 0.53 |  | 1061.0 | (707.0–1780.0 | 753.0 | (459.5–1267.8) | 0.10 |
| Weight, kg | 69.1 | (65.4–81.5) | 67.1 | (62.4–78.1) | 0.34 |  | 68.1 | (64.5–81.5) | 68.0 | (62.4  –78.4) | 0.57 |  | 68.6 | (63.9–77.0) | 67.6 | (64.9–82.5) | 0.55 |
| Δ weight, kg | -0.2 | (-1.2–0.8) | -0.40 | (-1.9–  0.8) | 0.67 |  | -0.2 | (-1.2–  0.8) | -0.6 | (-1.9–0.5) | 0.40 |  | 0.1 | (-1.2–  1.6) | -0.8 | (-2.3–  0.2) | 0.061 |
| BMI, kg/m^2^ | 27.8 | (24.1–29.8) | 26.2 | (24.1–28.2) | 0.42 |  | 26.8 | (24.1–30.0) | 25.9 | (24.0  –28.2) | 0.24 |  | 26.1 | (24.0–28.5) | 26.6 | (24.4–33.3) | 0.19 |
| Total body fat mass, kg | 33.7 | (25.7–41.4) | 30.6 | (25.0–38.3) | 0.47 |  | 33.7 | (27.0–42.3) | 29.9 | (25.0–36.3) | 0.072 |  | 22.8 | (16.6–26.2) | 22.4 | (16.3–33.2) | 0.34 |
| Δ total body fat mass, kg | -0.6 | (-1.6–  0.7) | -0.5 | (-1.8–0.4) | 0.80 |  | -0.6 | (-1.8–  3.0) | -0.95 | (-5.6–  1.2) | 0.46 |  | -0.6 | (-1.8–  0.4) | -0.5 | (-1.7–0.6) | 0.73 |
| Body fat percentage, % | 33.7 | (25.7–41.4) | 30.6 | (25.0–38.3) | 0.47 |  | 33.7 | (27.0–42.3) | 29.9 | (25.0–36.3) | 0.073 |  | 30.6 | (25.4–38.3) | 33.1 | (26.5–42.9) | 0.31 |
| Δ body fat percentage, % | -0.6 | (-2.5–1.2) | -0.7 | (-1.8–  0.4) | 0.31 |  | -0.6 | (-1.8–  0.4) | -1.0 | (-5.6–1.2) | 0.46 |  | -0.8 | (-2.7–  0.5) | 0.1 | (-1.1–  0.9) | 0.18 |
| Muscle quality score | 58.0 | (46.5–67.5) | 47.5 | (41.0–58.0) | 0.051 |  | 54.0 | (44.0–65.3) | 47.5 | (38.0–58.0) | 0.14 |  | 52.0 | (41.0–  63.0) | 51.0 | (44.0–64.0) | 0.89 |
| Δ muscle quality score | 0 | (-3.5–10.0) | 5.0 | (-4.0–11.0) | 0.35 |  | 2.0 | (-3.3–10.0) | 4.5 | (-5.0–9.0) | 0.68 |  | 4.0 | (-1.0–  10.0) | -1.0 | (-5.3–10.3) | 0.32 |
| SMI, kg/m^2^ | 7.70 | (7.33–8.15) | 7.95 | (7.00–8.50) | 0.75 |  | 7.70 | (7.10–8.13) | 8.15 | (7.30–8.80) | 0.23 |  | 7.80 | (7.10–  8.40) | 7.80 | (6.98–8.43) | 0.89 |
| Δ SMI, kg/m^2^ | 0.10 | (-0.10–0.40) | 0 | (-0.10–0.20) | 0.38 |  | 0.10 | (-0.10–0.30) | -0.05 | (-0.10–0.40) | 0.76 |  | 0.10 | (-0.10–0.40) | -0.10 | (-0.13–0.13) | 0.047* |
| SMM |  |  |  |  |  |  |  |  |  |  |  |  |  |  |  |  |  |
| Whole body, kg | 47.0 | (40.5–49.5) | 45.1 | (36.6 –51.3) | 0.92 |  | 42.0 | (37.5–48.8) | 47.9 | (39.3–52.0) | 0.24 |  | 47.6 | (39.3–50.2) | 43.1 | (36.3–49.7) | 0.41 |
| Δ whole body, kg | 0 | (-0.8–1.5) | -0.1 | (-0.9–  0.6) | 0.35 |  | 0 | (-0.4–  1.0) | -0.2 | (-0.9–1.1) | 0.59 |  | 0.5 | (-0.7–  1.6) | -0.3 | (-0.9–  0.3) | 0.15 |
| Trunk, kg | 25.6 | (21.1–26.8) | 25.0 | (19.4–27.6) | 0.84 |  | 21.7 | (20.1–26.3) | 26.5 | (20.9–28.4) | 0.30 |  | 25.6 | (21.2–27.6) | 21.7 | (19.8–26.8) | 0.48 |
| Δ trunk, kg | 0.1 | (-0.3–  0.4) | -0.1 | (-0.3–  0.2) | 0.35 |  | -0.1 | (-0.3–  0.3) | -0.1 | (-0.3–0.3) | 0.48 |  | 0.1 | (-0.3–  0.3) | -0.1 | (-0.3–  0.2) | 0.35 |
| Left leg, kg | 8.3 | (7.5–  8.9) | 8.2 | (6.5–  9.3) | 0.83 |  | 8.2 | (6.9–  8.8) | 8.3 | (7.8–9.30) | 0.44 |  | 8.4 | (7.5–  9.2) | 7.8 | (6.7–  8.6) | 0.31 |
| Δ left leg, kg | 0.1 | (-2.0–  0.2) | -0.1 | (-0.3–  3.0) | 0.49 |  | 0 | (-0.2–  0.2) | -0.1 | (-0.3–0.3) | 0.62 |  | 0.1 | (-0.2–  0.4) | -0.1 | (-0.3–  0.2) | 0.077 |
| Right leg, kg | 8.0 | (7.6–  8.7) | 8.0 | (6.7–  9.2) | 0.95 |  | 8.0 | (6.8–  8.9) | 8.3 | (7.5 –9.7) | 0.42 |  | 8.2 | (7.70–9.10) | 7.6 | (6.7–  9.2) | 0.38 |
| Δ right leg, kg | 0 | (-0.1–  0.30) | -0.1 | (-0.2–  0.2) | 0.33 |  | 0 | (-0.1–  0.3) | -0.1 | (-0.2–0.2) | 0.46 |  | 0.1 | (-0.1–  0.4) | -0.1 | (-0.2–  0) | 0.036***** |
| Systolic blood pressure, mmHg | 128.0 | (117.0–139.3) | 120.0 | (110.0–  125.0) | 0.020***** |  | 123.0 | (116.3–135.5) | 121.5 | (110.0–126.0) | 0.15 |  | 121.5 | (114.0–131.0) | 124.0 | (110.8–131.0) | 0.95 |
| Δ systolic blood pressure, mmHg | -1.0 | (-12.5–  5.3) | -0.5 | (-10.0–8.0) | 0.46 |  | 0 | (-10.5–4.3) | -2.0 | (-10.0– 12.0) | 0.62 |  | 2.0 | (-10.0–  6.0) | -4.0 | (-11.3–20.3) | 0.45 |
| Diastolic blood pressure, mmHg | 77.0 | (70.0–  82.5) | 72.0 | (66.0–80.0) | 0.096 |  | 76.0 | (70.0  –82.0 | 75.5 | (70.0–80.0) | 0.64 |  | 76.0 | (70.0–  80.0) | 75.0 | (68.5–105.0) | 0.93 |
| Δ diastolic blood pressure, mmHg | 2.0 | (-10.0–  7.3) | 4.5 | (-2.0–  9.0) | 0.10 |  | 3.0 | (-8.0–  8.0) | 4.0 | (-2.0–9.0) | 0.41 |  | 4.0 | (-6.0–11.0) | 2.0 | (-6.5–7.3) | 0.18 |
| HbA1c, % | 7.10 | (6.88–  7.73) | 7.05 | (6.70–7.70) | 0.34 |  | 7.10 | (6.88–7.63) | 7.10 | (6.60–7.70) | 0.66 |  | 7.1 | (6.70–7.70) | 7.2 | (6.88–7.70) | 0.70 |
| Δ HbA1c, % | 0.20 | (0.08–  0.53) | 0.10 | (-0.20–0.20) | 0.15 |  | 0.10 | (-0.10–0.43) | 0.20 | (-0.20–0.30) | 0.98 |  | 0.10 | (-0.10–0.30) | 0.2 | (-0.23–0.60) | 0.97 |
| eGFR, mL/min/1.73 m^2^ | 68.0 | (57.5–  78.3) | 66.0 | (59.0–79.0) | 0.98 |  | 68.0 | (60.3–82.3) | 64.0 | (54.0–71.0) | 0.22 |  | 65.0 | (53.0–71.0) | 71.0 | (64.0–80.0) | 0.030***** |
| Δ eGFR, mL/min/1.73 m^2^ | 1.0 | (-4.0–  5.3) | -2.0 | (-6.0–  1.0) | 0.20 |  | 1.0 | (-5.0–  5.0) | -2.0 | (-7.0–1.0) | 0.25 |  | 0.5 | (-4.0–  3.0) | -2.0 | (-5.0–3.5) | 0.65 |
| LDL-C, mg/dL | 97.0 | (85.5–  107.3) | 99.0 | (82.0–117.0) | 0.99 |  | 97.0 | (83.0–111.8) | 99.5 | (88.0–114.0) | 0.66 |  | 93.0 | (83.0–111.0) | 100.0 | (87.8–122.8) | 0.19 |
| Δ LDL-C, mg/dL | 12.0 | (-9.8–  18.3) | 0.5 | (-10.0–11.0) | 0.13 |  | 8.0 | (-9.0–  15.8) | 0.5 | (-10.0–11.0) | 0.23 |  | 7.5 | (-9.0–  18.0) | 6.0 | (-10.0–11.3) | 0.45 |
| HDL-C, mg/dL | 58.0 | (49.0–  64.5) | 47.5 | (40.0–57.0) | 0.025* |  | 55.0 | (45.5–61.3) | 53.0 | (43.0–60.0) | 0.66 |  | 53.5 | (46.0–  60.0) | 55.0 | (38.8–62.3) | 0.98 |
| Δ HDL-C, mg/dL | 2.0 | (-3.3–  5.3) | 4.0 | (-2.0–10.0) | 0.30 |  | 2.0 | (-4.0–  6.8) | 4.5 | (-1.0–  7.0) | 0.24 |  | 3.0 | (-3.0–  7.0) | 2.0 | (-2.5–6.8) | 0.83 |
| Casual triglycerides, mg/dL | 137.0 | (92.0–  191.8) | 154.5 | (111.0–177.0) | 0.47 |  | 148.0 | (104.0–183.5) | 127.5 | (103.0–177.0) | 0.70 |  | 124.5 | (97.0–191.0) | 155.0 | (111.8–179.5) | 0.28 |
| Δ casual triglyceride, mg/dL | -7.0 | (-47.5–  14.0) | -12.0 | (71.0) | 0.77 |  | -8.0 | (-46.6–18.0) | -11.0 | (-31.0–29.0) | 0.75 |  | -12.0 | (-45.0–13.0) | 5.0 | (-41.3–27.3) | 0.42 |
| UACR, mg/gCre | 19.5 | (9.5–  35.5) | 13.0 | (7.8–  51.8) | 0.61 |  | 19.0 | (8.5–  42.5) | 12.0 | (6.8–  55.3) | 0.48 |  | 17.0 | (7.5–  45.5) | 18.0 | (8.0–41.8) | 0.66 |
| Δ UACR, mg/gCre | 2.0 | (-5.3–  13.7) | 0 | (-13.8–8.5) | 0.37 |  | 97.0 | (83.0–111.8) | 99.5 | (88.0–114.0) | 0.66 |  | 93.0 | (83.0–111.0) | 100.0 | (87.8–122.8) | 0.19 |

Continuous variables are presented as median (interquartile range). Categorial variables were presented as n (%). Deita (Δ) represents the subtraction of pre-interval walking training (IWT) value from post-IWT value of each item. *Statistically significant (p <0.05) according to the Mann-Whitney U test. F/w, the maximum ground reaction force based on weight; RFD/w, rate of force development based on weight; BIS, balance index score; BMI, body mass index; DM, diabetes mellitus; SMI, skeletal muscle index; SMM, skeletal muscle mass; eGFR, estimated glomerular filtration rate; LDL-C, low density lipoprotein cholesterol; HDL-C, high density lipoprotein cholesterol; UACR, urinary albumin creatinine rate.
