# Supplementary material for: Interval walking training as a potential contributor to motor function improvement in adults with type 2 diabetes mellitus: a retrospective analysis
Source: Front Endocrinol (Lausanne). 2025 Jun 9;16:1544831. doi: 10.3389/fendo.2025.1544831 (PMC12183035; doi:10.3389/fendo.2025.1544831)
Supplement: Supplementary file 3 [file Table3.docx]

**Supplementary Table 3** Significant factors associated with changes in motor function in older and non-older age groups

1. **Δ F/w, kgf/kg**

| **Independent variables** | **Older age group**  **(N = 24)** | |  | **Non-older age group**  **(N = 27)** | |
| --- | --- | --- | --- | --- | --- |
|  | **ρ** | **p** |  | **ρ** | **p** |
| Age, years | 0.397 | 0.055 |  | -0.227 | 0.25 |
| Duration of DM, years | -0.035 | 0.87 |  | -0.100 | 0.62 |
| Total fast walking time, min | -0.039 | 0.86 |  | 0.047 | 0.82 |
| Weight, kg | -0.077 | 0.72 |  | 0.065 | 0.75 |
| Δ Weight, kg | 0.147 | 0.49 |  | -0.018 | 0.93 |
| BMI, kg/m^2^ | -0.277 | 0.19 |  | 0.316 | 0.11 |
| Total body fat mass, kg | -0.261 | 0.22 |  | 0.303 | 0.12 |
| Δ total body fat mass, kg | 0.147 | 0.49 |  | 0.098 | 0.63 |
| Body fat percentage, % | -0.261 | 0.22 |  | 0.408 | 0.035* |
| Δ body fat percentage, % | 0.164 | 0.45 |  | 0.174 | 0.39 |
| Muscle quality score | 0.005 | 0.98 |  | 0.638 | <0.001* |
| Δ muscle quality score | -0.137 | 0.52 |  | -0.044 | 0.83 |
| SMI, kg/m^2^ | 0.063 | 0.77 |  | -0.032 | 0.87 |
| Δ SMI, kg/m^2^ | 0.091 | 0.67 |  | -0.035 | 0.86 |
| SMM |  |  |  |  |  |
| Whole body, kg | 0.102 | 0.64 |  | -0.155 | 0.44 |
| Δ whole body, kg | 0.084 | 0.70 |  | -0.017 | 0.93 |
| Trunk, kg | 0.032 | 0.88 |  | -0.077 | 0.70 |
| Δ trunk, kg | 0.145 | 0.50 |  | 0.103 | 0.61 |
| Left leg, kg | 0.177 | 0.41 |  | -0.068 | 0.74 |
| Δ left leg, kg | 0.148 | 0.49 |  | -0.050 | 0.80 |
| Right leg, kg | 0.128 | 0.55 |  | -0.086 | 0.67 |
| Δ right leg, kg | 0.093 | 0.67 |  | -0.098 | 0.63 |
| Systolic blood pressure, mmHg | 0.548 | 0.006* |  | 0.355 | 0.070 |
| Δ systolic blood pressure, mmHg | -0.294 | 0.16 |  | 0.117 | 0.56 |
| Diastolic blood pressure, mmHg | 0.199 | 0.35 |  | 0.250 | 0.21 |
| Δ diastolic blood pressure, mmHg | -0.266 | 0.21 |  | -0.208 | 0.30 |
| HbA1c, % | -0.057 | 0.79 |  | 0.005 | 0.82 |
| Δ HbA1c, % | 0.305 | 0.15 |  | 0.303 | 0.12 |
| eGFR, mL/min/1.73 m^2^ | 4E-4 | 0.99 |  | -0.018 | 0.93 |
| ΔeGFR, mL/min/1.73 m^2^ | 0.242 | 0.25 |  | 0.004 | 0.98 |
| LDL-C, mg/dL | -0.313 | 0.14 |  | 0.319 | 0.10 |
| Δ LDL-C, mg/dL | 0.146 | 0.50 |  | 0.398 | 0.040* |
| HDL-C, mg/dL | 0.355 | 0.089 |  | 0.299 | 0.13 |
| Δ HDL-C, mg/dL | -0.234 | 0.27 |  | -0.033 | 0.87 |
| Casual triglycerides, mg/dL | -0.381 | 0.066 |  | 0.151 | 0.45 |
| Δ casual triglycerides, mg/dL | 0.020 | 0.93 |  | -0.014 | 0.95 |
| UACR, mg/gCre | 0.076 | 0.73 |  | 0.109 | 0.60 |
| Δ UACR, mg/gCre | 0.053 | 0.81 |  | 0.071 | 0.75 |
| F/w | -0.344 | 0.10 |  | -0.313 | 0.11 |
| RFD/w | -0.265 | 0.212 |  | -0.185 | 0.35 |
| Δ RFD/w | 0.607 | 0.002* |  | 0.793 | <0.001* |
| BIS | -0.192 | 0.368 |  | 0.049 | 0.81 |
| Δ BIS | 0.197 | 0.356 |  | -0.005 | 0.98 |

**(b) Δ RFD/w, kgf/s/kg**

| **Independent variables** | **Older age group**  **(N = 24)** | |  | **Non-older age group**  **(N = 27)** | |
| --- | --- | --- | --- | --- | --- |
|  | **ρ** | **p** |  | **ρ** | **p** |
| Age, years | 0.339 | 0.11 |  | -0.381 | 0.049* |
| Duration of DM, years | 0.126 | 0.57 |  | -0.253 | 0.20 |
| Total fast walking time, min | 0.169 | 0.43 |  | -0.141 | 0.48 |
| Weight, kg | -0.196 | 0.36 |  | 0.306 | 0.12 |
| Δ weight, kg | 0.119 | 0.58 |  | 0.051 | 0.80 |
| BMI, kg/m^2^ | -0.245 | 0.25 |  | 0.423 | 0.028* |
| Total body fat mass, kg | -0.099 | 0.65 |  | 0.457 | 0.017* |
| Δ total body fat mass, kg | -0.014 | 0.95 |  | 0.100 | 0.62 |
| Body fat percentage, % | -0.034 | 0.87 |  | 0.442 | 0.021* |
| Δ body fat percentage, % | 0.004 | 0.98 |  | 0.163 | 0.42 |
| Muscle quality score | -0.101 | 0.64 |  | 0.514 | 0.006* |
| Δ muscle quality score | 0.223 | 0.29 |  | -0.073 | 0.72 |
| SMI, kg/m^2^ | -0.314 | 0.14 |  | 0.154 | 0.44 |
| Δ SMI, kg/m^2^ | 0.199 | 0.35 |  | 0.016 | 0.94 |
| SMM |  |  |  |  |  |
| Whole body, kg | -0.266 | 0.21 |  | 0.021 | 0.92 |
| Δ whole body, kg | 0.258 | 0.22 |  | 0.062 | 0.76 |
| Trunk, kg | -0.368 | 0.077 |  | 0.026 | 0.90 |
| Δ trunk, kg | 0.375 | 0.071 |  | 0.164 | 0.41 |
| Left leg, kg | -0.192 | 0.37 |  | 0.152 | 0.45 |
| Δ left leg, kg | 0.263 | 0.21 |  | -0.064 | 0.75 |
| Right leg, kg | -0.196 | 0.36 |  | 0.124 | 0.54 |
| Δ right leg, kg | 0.215 | 0.31 |  | 0.008 | 0.97 |
| Systolic blood pressure, mmHg | 0.395 | 0.056 |  | 0.214 | 0.28 |
| Δ systolic blood pressure, mmHg | -0.322 | 0.13 |  | 0.029 | 0.89 |
| Diastolic blood pressure, mmHg | -0.193 | 0.37 |  | 0.213 | 0.29 |
| Δ diastolic blood pressure, mmHg | -0.034 | 0.87 |  | -0.223 | 0.26 |
| HbA1c, % | -0.132 | 0.54 |  | 0.171 | 0.39 |
| Δ HbA1c, % | 0.206 | 0.33 |  | 0.133 | 0.51 |
| eGFR, mL/mim/1.73 m^2^ | -0.036 | 0.87 |  | -0.012 | 0.95 |
| Δ eGFR, mL/mim/1.73 m^2^ | 0.259 | 0.22 |  | 0.352 | 0.072 |
| LDL-C, mg/dL | -0.225 | 0.29 |  | 0.152 | 0.45 |
| Δ LDL-C, mg/dL | 0.077 | 0.72 |  | 0.244 | 0.22 |
| HDL-C, mg/dL | 0.104 | 0.63 |  | 0.181 | 0.36 |
| Δ HDL-C, mg/dL | -0.448 | 0.028* |  | 0.002 | 0.99 |
| Casual triglycerides, mg/dL | -0.069 | 0.75 |  | 0.138 | 0.49 |
| Δ casual triglycerides, mg/dL | 0.132 | 0.54 |  | -0.089 | 0.66 |
| UACR, mg/gCre | 0.146 | 0.50 |  | -0.017 | 0.93 |
| Δ UACR, mg/gCre | -0.082 | 0.71 |  | 0.049 | 0.83 |
| F/w | -0.334 | 0.11 |  | -0.260 | 0.19 |
| RFD/w | -0.414 | 0.044* |  | -0.398 | 0.040* |
| BIS | 0.020 | 0.925 |  | 0.077 | 0.70 |
| Δ BIS | -0.094 | 0.661 |  | 0.032 | 0.87 |

**(c) Δ BIS**

| **Independent variables** | **Older age group**  **(N = 24)** | |  | **Non-older age group**  **(N = 27)** | | | |
| --- | --- | --- | --- | --- | --- | --- | --- |
|  | **ρ** | **p** |  | **ρ** | | **p** | |
| Age, years | 0.331 | 0.11 |  | 0.050 | | 0.80 | |
| Duration of DM, years | -0.455 | 0.029* |  | 0.125 | | 0.53 | |
| Total fast walking time, min | 0.151 | 0.48 |  | 0.356 | | 0.067 | |
| Weight, kg | 0.150 | 0.49 |  | -0.158 | | 0.43 | |
| Δ weight, kg | 0.434 | 0.034* |  | 0.137 | | 0.50 | |
| BMI, kg/m^2^ | -0.094 | 0.66 |  | -0.308 | | 0.12 | |
| Total body fat mass, kg | 0.0002 | 1.0 |  | -0.232 | | 0.24 | |
| Δ total body fat mass, kg | 0.115 | 0.59 |  | -0.161 | | 0.42 | |
| Body fat percentage, % | -0.167 | 0.43 |  | -0.229 | | 0.25 | |
| Δ body fat percentage, % | -0.089 | 0.68 |  | -0.282 | | 0.15 | |
| Muscle quality score | 0.145 | 0.50 |  | 0.007 | 0.97 | |  |
| Δ muscle quality score | -0.218 | 0.31 |  | 0.280 | 0.16 | |  |
| SMI, kg/m^2^ | 0.377 | 0.070 |  | -0.116 | 0.57 | |  |
| Δ SMI, kg/m^2^ | 0.216 | 0.31 |  | 0.348 | 0.075 | |  |
| SMM |  |  |  |  |  | |  |
| Whole body, kg | 0.461 | 0.023* |  | 0.001 | 1.00 | |  |
| Δ whole body, kg | 0.150 | 0.48 |  | 0.322 | 0.10 | |  |
| Trunk, kg | 0.273 | 0.20 |  | 0.093 | 0.64 | |  |
| Δ trunk, kg | 0.104 | 0.62 |  | 0.118 | 0.56 | |  |
| Left leg, kg | 0.491 | 0.015* |  | -0.047 | 0.81 | |  |
| Δ left leg, kg | 0.230 | 0.28 |  | 0.202 | 0.31 | |  |
| Right leg, kg | 0.445 | 0.029* |  | -0.051 | 0.80 | |  |
| Δ right leg, kg | 0.246 | 0.25 |  | 0.408 | 0.035* | |  |
| Systolic blood pressure, mmHg | 0.096 | 0.66 |  | 0.011 | 0.96 | |  |
| Δ systolic blood pressure, mmHg | -0.136 | 0.53 |  | 0.137 | 0.50 | |  |
| Diastolic blood pressure, mmHg | 0.398 | 0.054 |  | -0.110 | 0.59 | |  |
| Δ diastolic blood pressure, mmHg | -0.209 | 0.33 |  | 0.308 | 0.12 | |  |
| HbA1c, % | -0.262 | 0.22 |  | 0.161 | 0.42 | |  |
| Δ HbA1c, % | 0.175 | 0.41 |  | 0.020 | 0.92 | |  |
| eGFR, mL/min/1.73 m^2^ | -0.439 | 0.032* |  | -0.227 | 0.25 | |  |
| Δ eGFR, mL/min/1.73 m^2^ | 0.208 | 0.33 |  | -0.014 | 0.94 | |  |
| LDL-C, mg/dL | -0.407 | 0.049* |  | -0.005 | 0.98 | |  |
| Δ LDL-C, mg/dL | 0.158 | 0.46 |  | 0.010 | 0.96 | |  |
| HDL-C, mg/dL | -0.260 | 0.22 |  | -0.115 | 0.57 | |  |
| Δ HDL-C, mg/dL | 0.180 | 0.40 |  | -0.032 | 0.88 | |  |
| Casual triglycerides, mg/dL | -0.136 | 0.53 |  | 0.117 | 0.56 | |  |
| Δ casual triglycerides, mg/dL | -0.014 | 0.95 |  | -0.236 | 0.24 | |  |
| UACR, mg/gCre | 0.030 | 0.89 |  | -0.049 | 0.82 | |  |
| Δ UACR, mg/gCre | 0.124 | 0.57 |  | -0.317 | 0.14 | |  |
| F/w | 0.010 | 0.96 |  | -0.065 | 0.75 | |  |
| RFD/w | 0.089 | 0.68 |  | -0.195 | 0.33 | |  |
| BIS | -0.614 | 0.001* |  | -0.715 | <0.001* | |  |

Delta (Δ) represents the subtraction of pre-IWT value from post-IWT value of each item. *Statistically significant (p <0.05) according to the Spearman’s rank correlation. F/w, the maximum ground reaction force based on weight; RFD/w, rate of force development based on weight; BIS, balance index score; DM, diabetes mellitus; BMI, body mass index; BMI, body mass index; HDL-C, high density lipoprotein cholesterol; eGFR, estimated glomerular filtration rate; SMI, skeletal muscle index; SMM, skeletal muscle mass; UACR, urinary albumin creatinine ratio.
